# Supplementary material for: Black indium oxide a photothermal CO2 hydrogenation catalyst
Source: Nat Commun. 2020 May 15;11:2432. doi: 10.1038/s41467-020-16336-z (PMC7229034; doi:10.1038/s41467-020-16336-z)
Supplement: Supplementary file 1 — SUPPLEMENTARY INFO [file 41467_2020_16336_MOESM1_ESM.pdf]

Supplementary Information

Black Indium Oxide a Photothermal CO_2 Hydrogenation Catalyst

Wang *et al.*

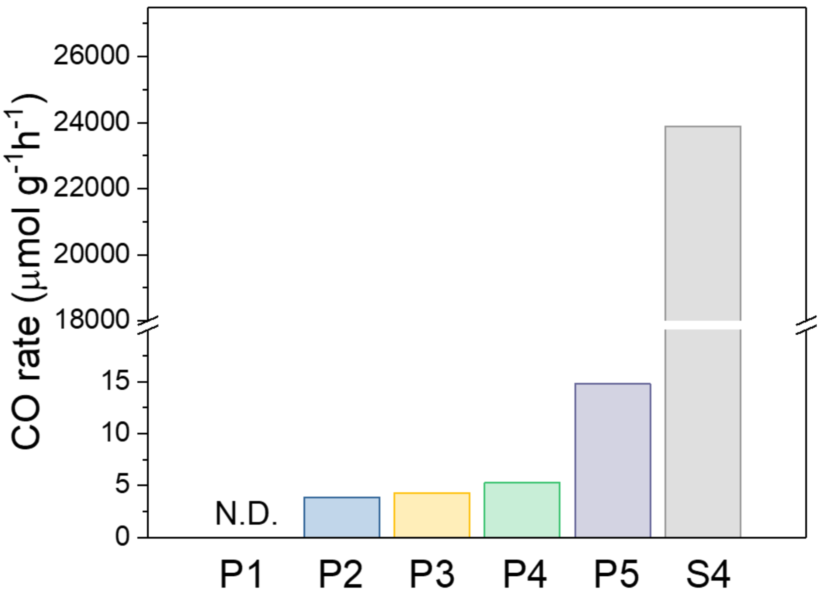

Supplementary Figure 1. Photothermal performance of typical In_2O_3 based photocatalysts from a batch reactor, including commercial In_2O_3 (P1), In_2O_3 synthesized from thermal decomposition of $In(NO)_3$ at 300 ˚C (P2), superstructure nanorod $In_2O_{3-x}(OH)_y$ (P3)[1], rhombohedral $In_2O_{3-x}(OH)_y$ nanocrystal (P4)[2], cubic $In_2O_{3-x}(OH)_y$ nanocrystal (P5)[1], and black indium oxide (S4).

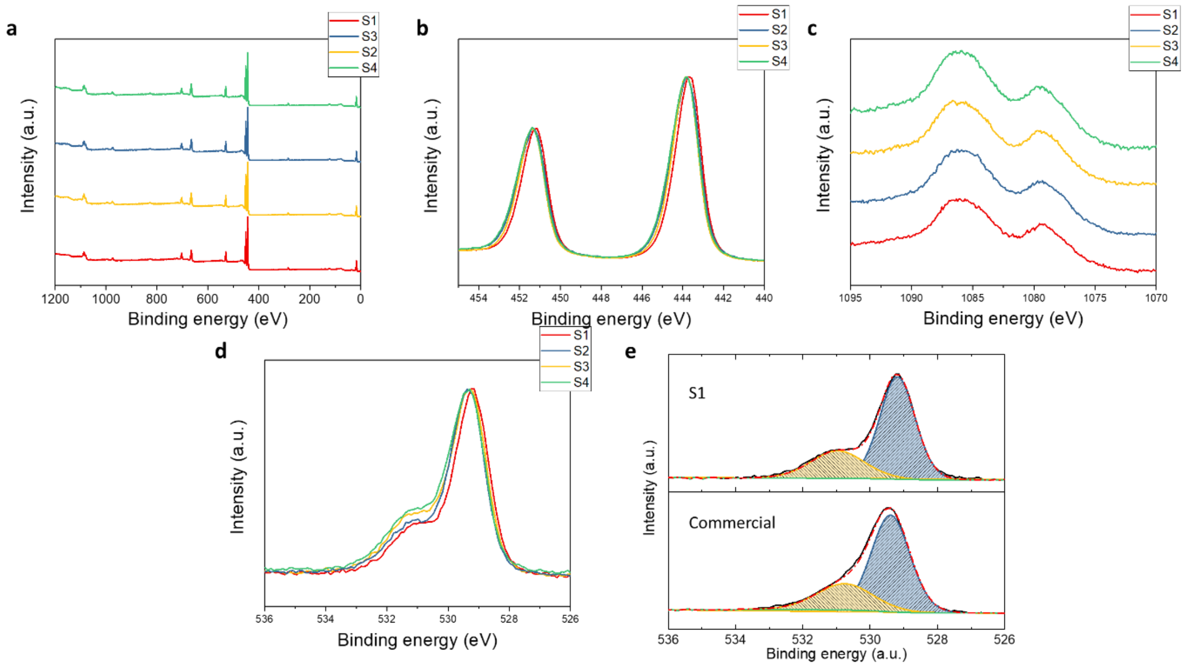

Supplementary Figure 2. (a) Wide scan of S1-S4. High resolution XPS spectrums of (b) In 3d, (c) Auger LMM of In and (d) O 1s for all samples. (e) High resolution XPS spectrum of O1s core level for S1 and commercial In_2O_3. The concentration of InO_{6-x} for commercial In_2O_3, S1 to S4 are 28.33%, 28.91%, 31.74%, 34.97% and 37.34%, respectively. Based on the very similar composition for S1 and commercial In_2O_3, the S1 is assumed to be In_2O_3.

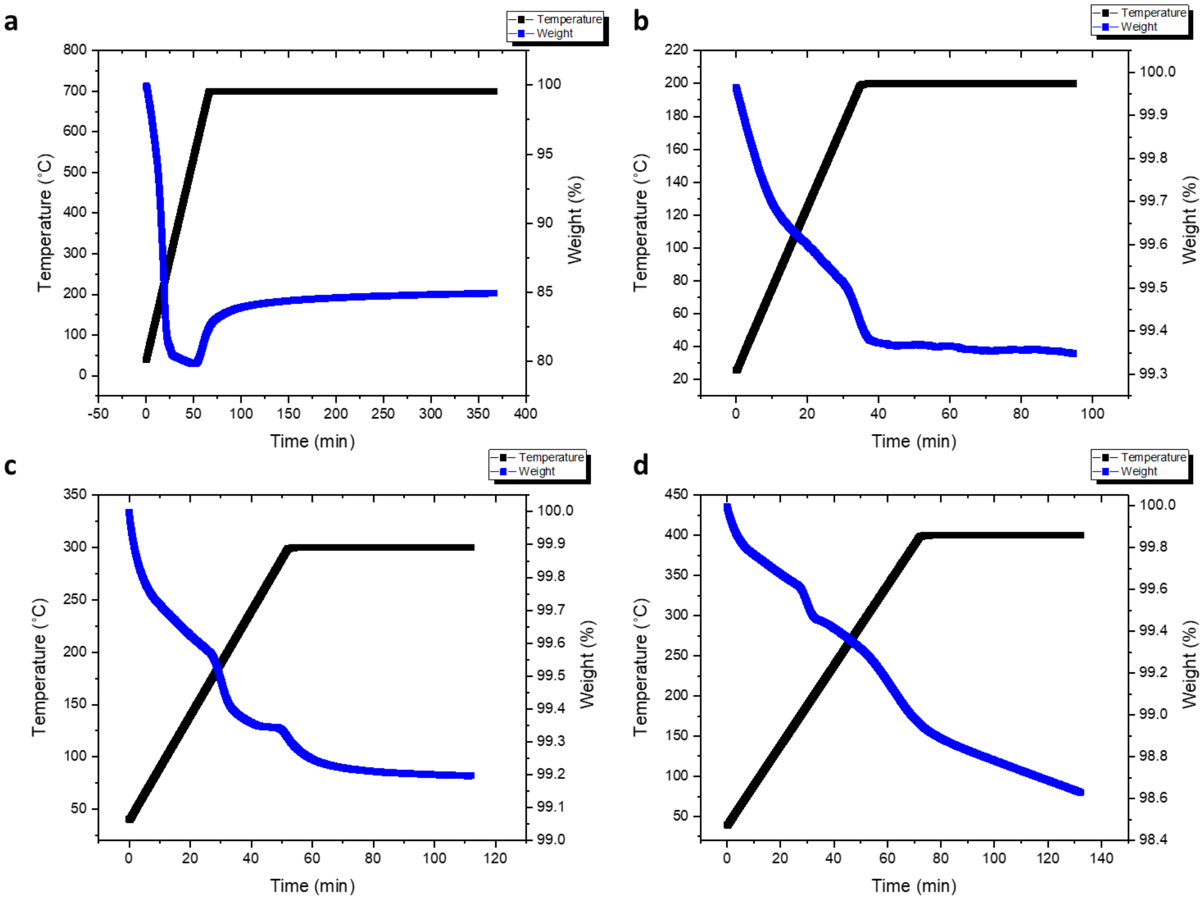

Supplementary Figure 3. TGA spectrum of simulated synthetic process of (a-d) S1 to S4. The overall process mainly includes fast dehydration, slow generation of oxygen vacancies and slow re-oxidation of the generated vacancies.

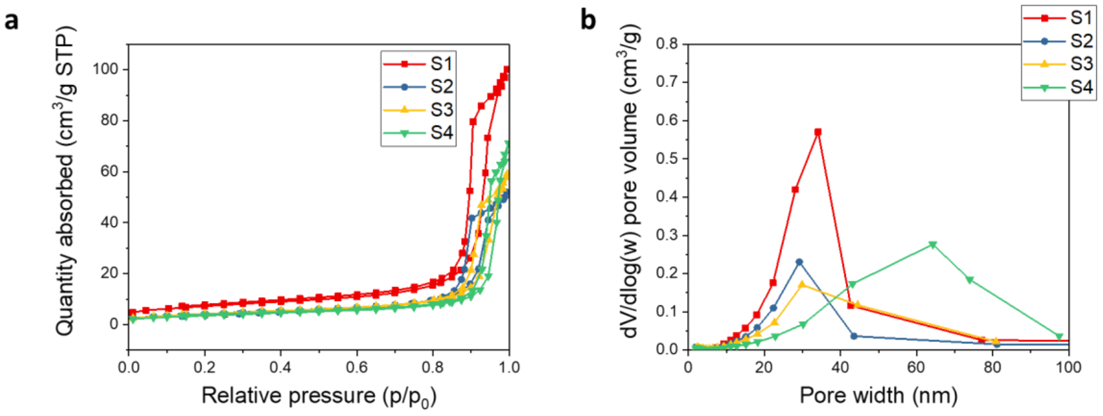

Supplementary Figure 4. (a) N_2 physisorption isotherms (adsorption-desorption branches), and (b) pore size distributions of all samples.

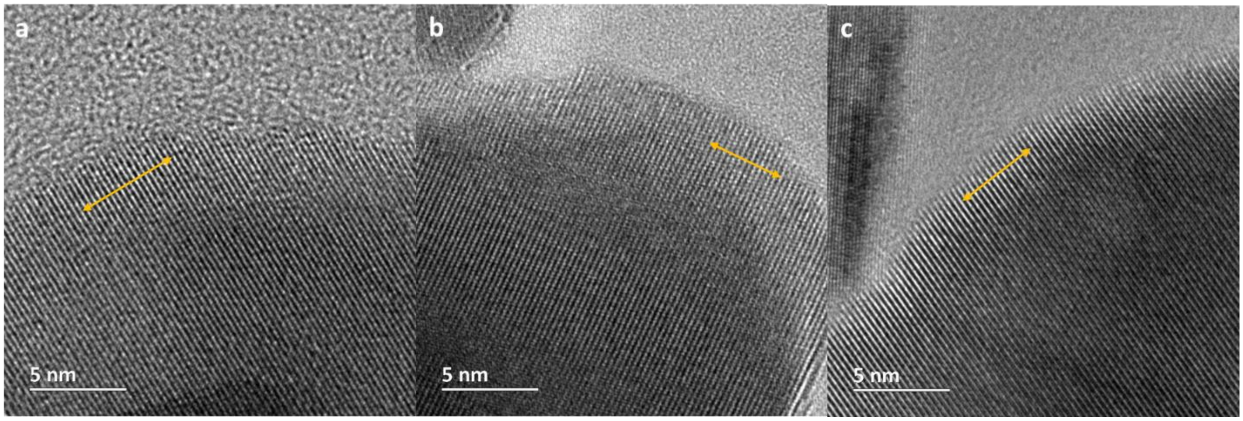

Supplementary Figure 5. HRTEM images of (a) S1, (b) S2 and (c) S3. The yellow arrows indicate the measured lattice spacing's with a value of ~0.292 nm.

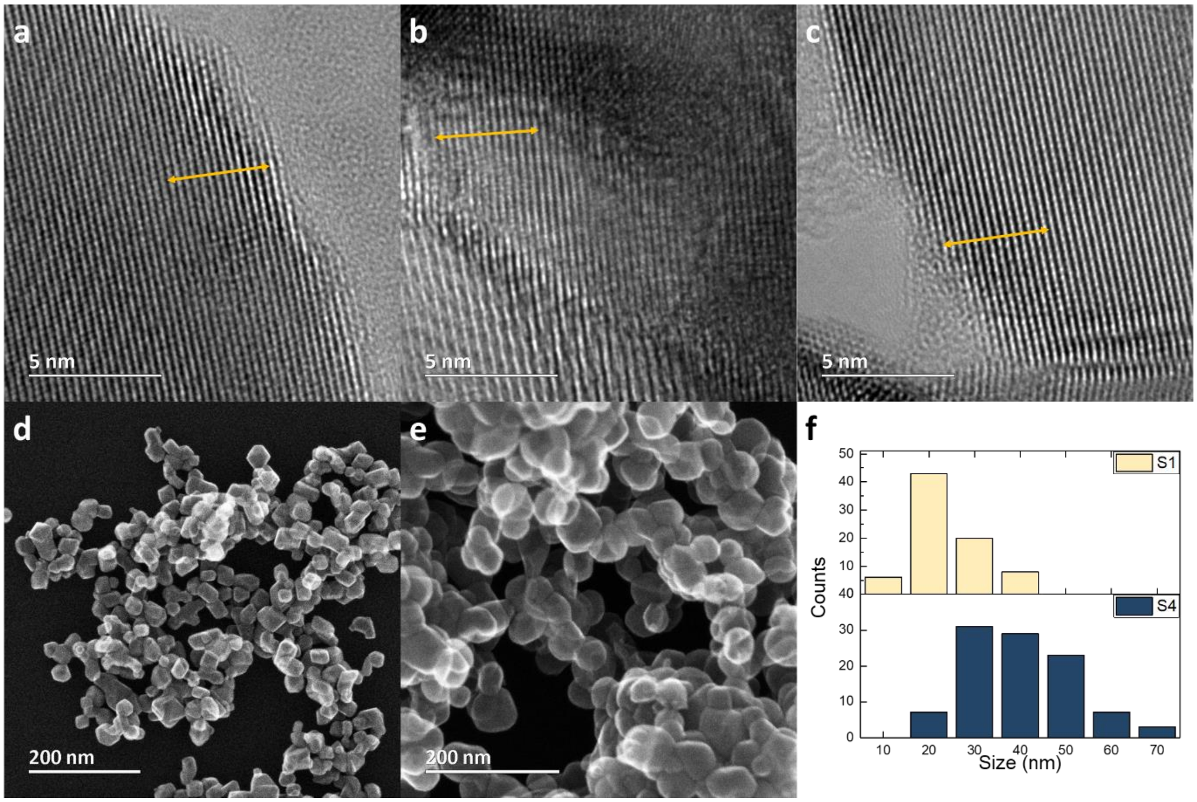

Supplementary Figure 6. High-resolution transmission electron microscopy images of (a-c) S4, with measured d spacing of 0.296 nm, 0.297 nm and 0.297 nm, respectively. STEM images of (d) S1 and (e) S4 with average particle sizes (f) of 28.8 nm and 44.9 nm, respectively.

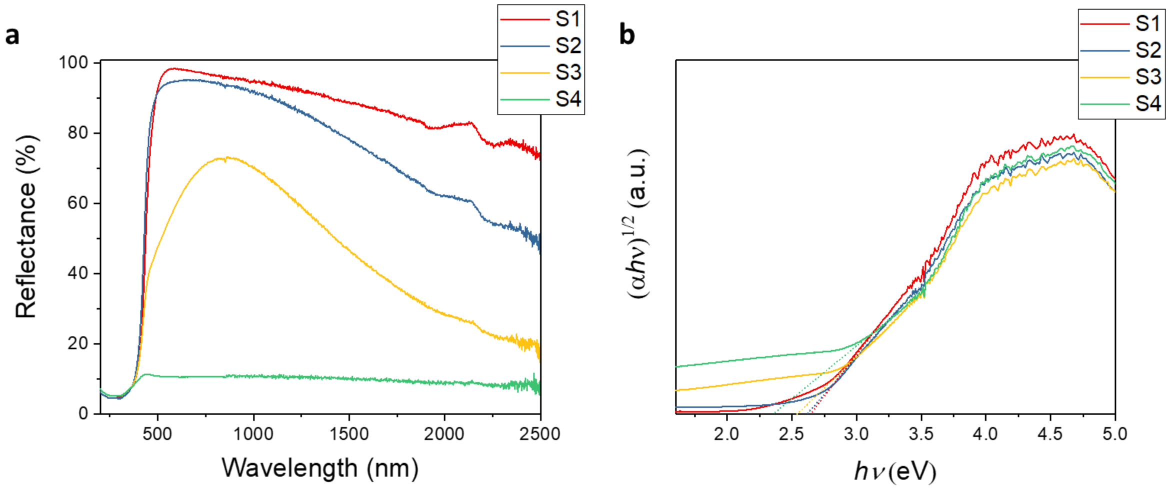

Supplementary Figure 7. (a) Optical reflectance spectra (UV-VIS-NIR) and (b) Tauc plot of Kubelka-Munk function for S1-S4 samples.

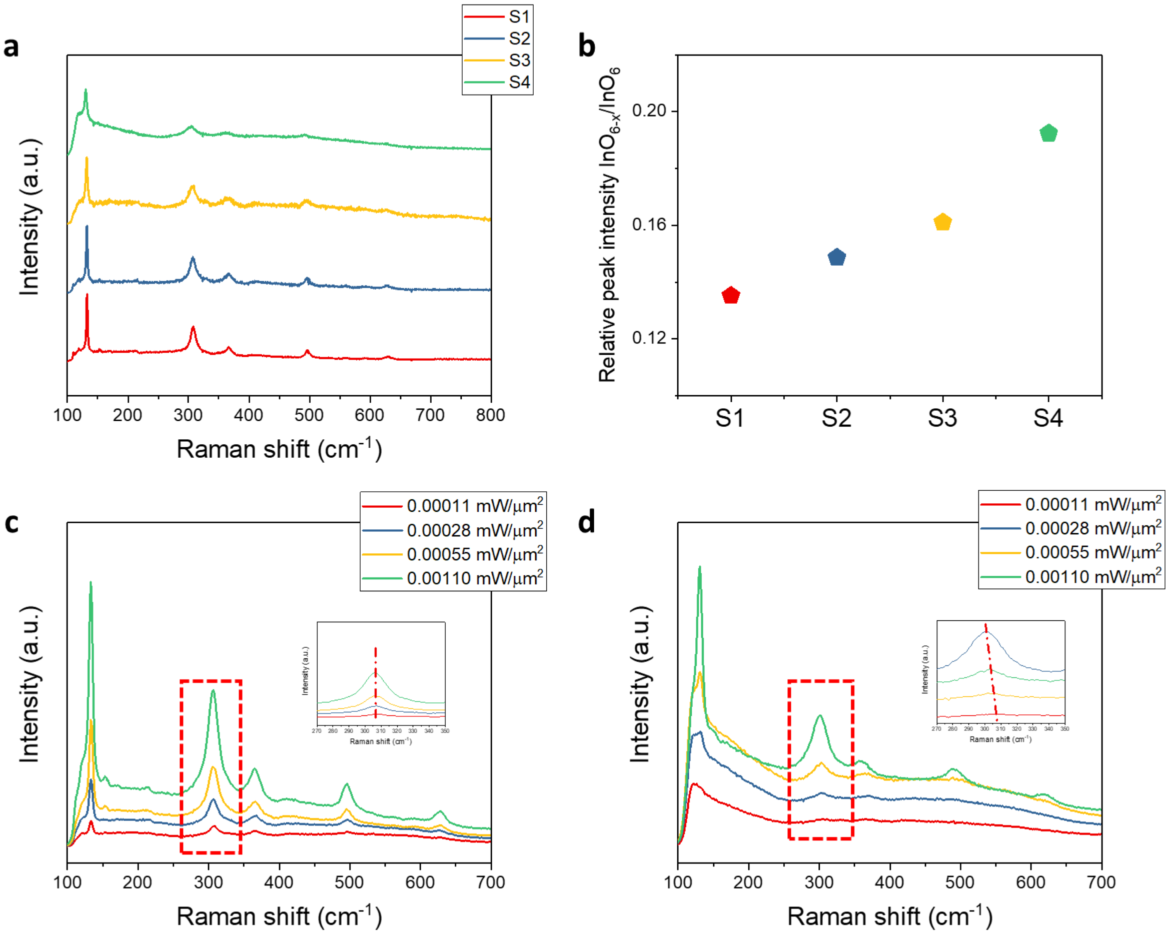

Supplementary Figure 8. (a) Raman spectra for S1-S4. The obtained full-width half-maximum of S1 to S4 are 3.24, 3.61, 4.82 and 6.21 cm^{-1}, respectively. (b) Ratio between InO_{6-x} and InO_6. Raman spectra with different beam intensities over S3 (c) and S4 (d).

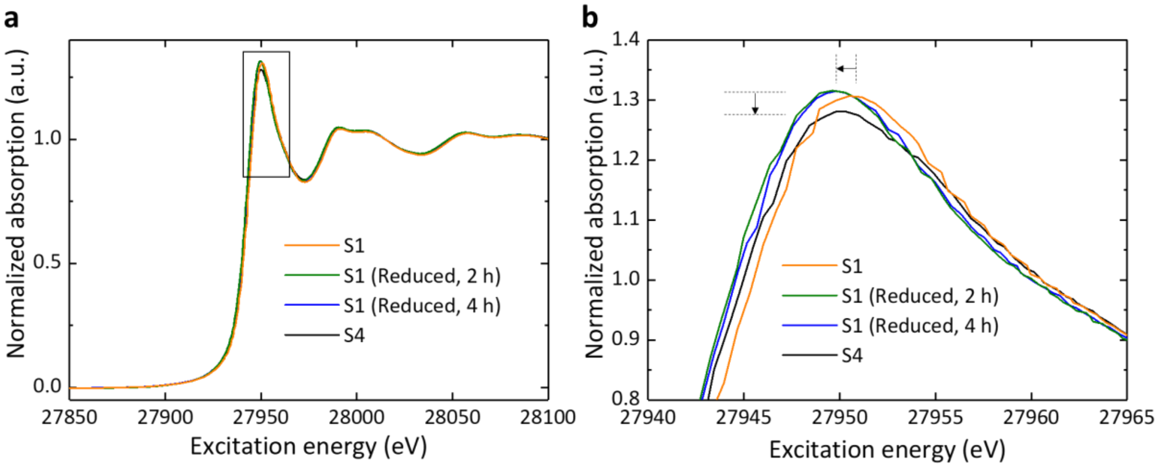

Supplementary Figure 9. Indium K-edge XANES spectra obtained from In_2O_3 samples reduced *in situ* (S1) and *ex situ* (S4). A shift of the white line peak to lower excitation energy is observed during *in situ* H_2 treatment. A reduction in amplitude is observed for the sample prepared *ex situ.*

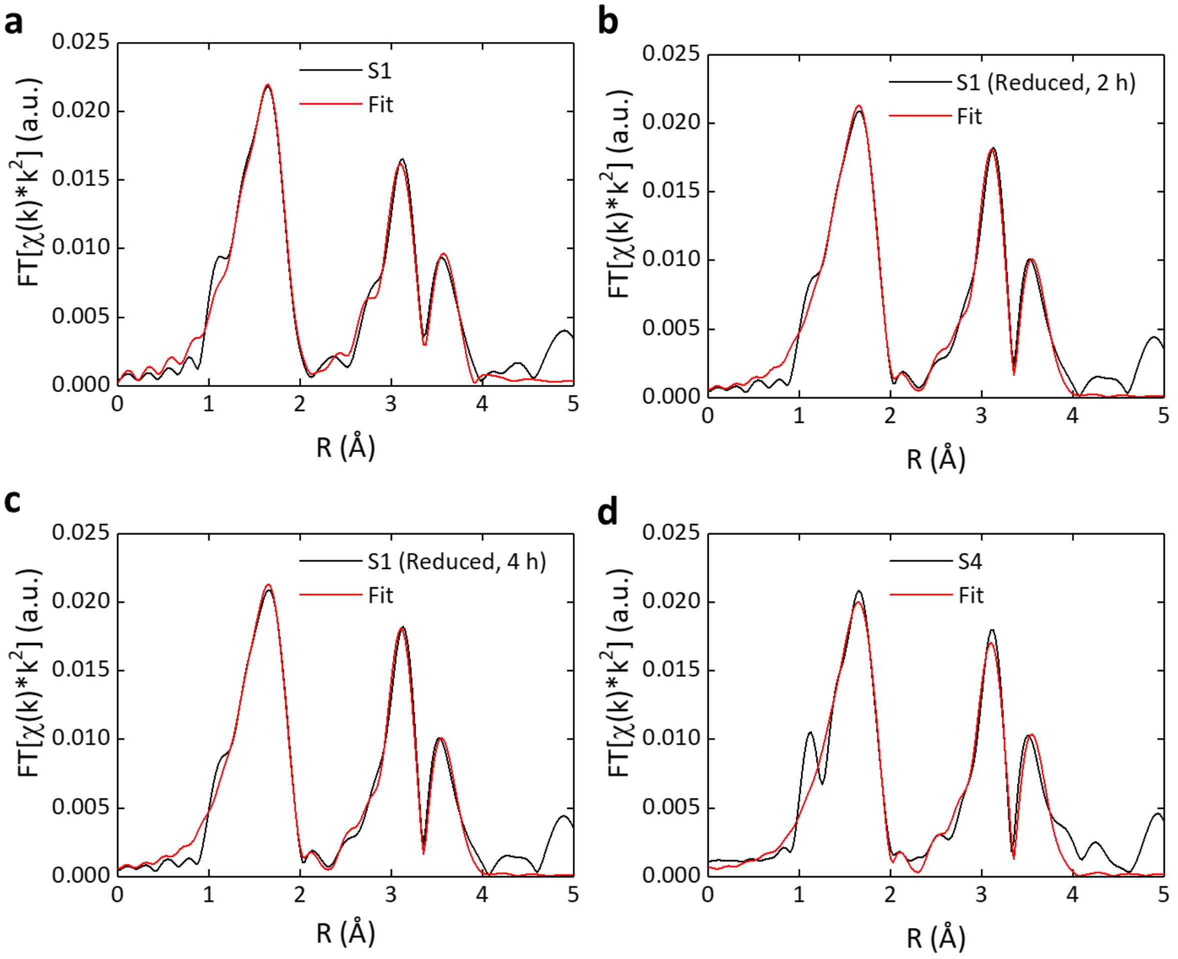

Supplementary Figure 10. Fourier-transformed In K-edge EXAFS spectra for samples reduced *in situ* (S1) and *ex situ* (S4). Close agreement between the experimental spectra (black) and fitted function (red) through to 4.0 Å indicates that no significant contributions remain unaccounted for.

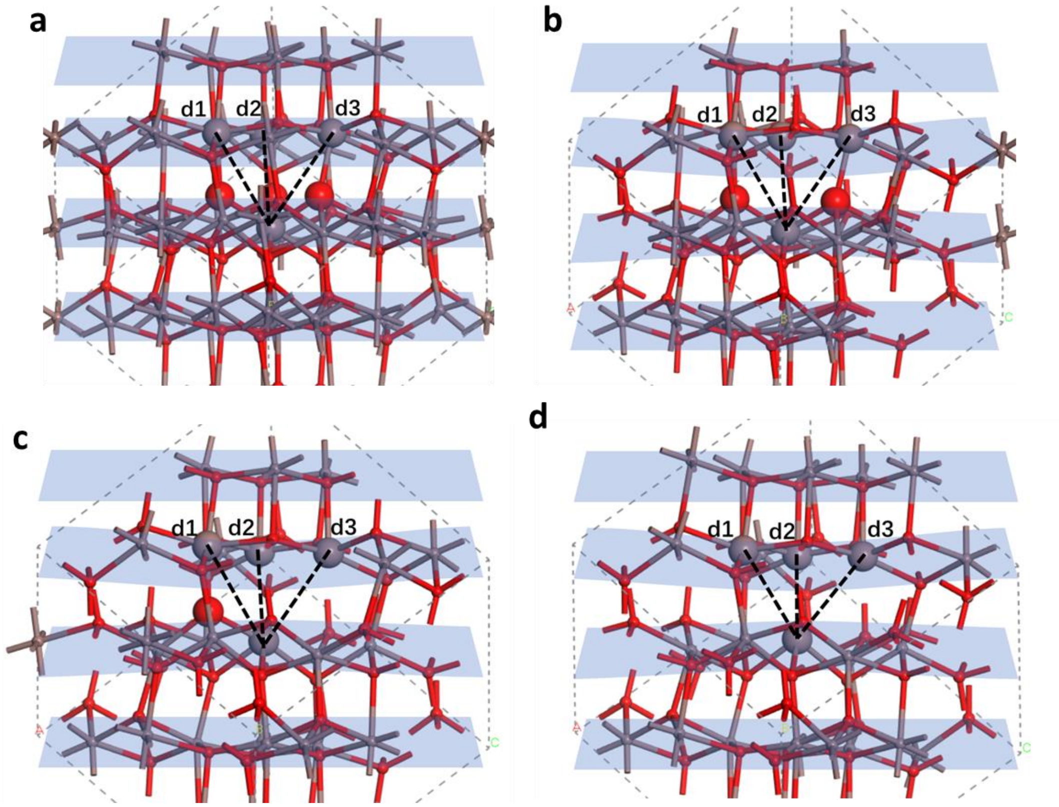

Supplementary Figure 11. (a) The simulated structure of an In_2O_3 cubic cell (b) with one O atom removed, (c) with two adjacent O atoms removed and (d) with three adjacent O atoms removed. Gray and red spheres indicate In and O atoms respectively. Larger spheres show atoms adjacent to vacancies. The shadows represent the plane of (222) facets, which are slightly distorted when vacancies are present. The d1, d2 and d3 lines indicate In-In distances that will shrink when oxygen is removed. The resulting simulated cells indicate the In-In distances are seen to decrease with increasing numbers of removed O atoms. Specifically, with removal of three O atoms, the resulting In-In distances are 7.5% smaller than those in the crystalline stoichiometric phase, inducing a localized shrinkage of the lattice and resulting in a possible expansion of the adjacent lattice, a deduction that agrees well with the experimental findings.

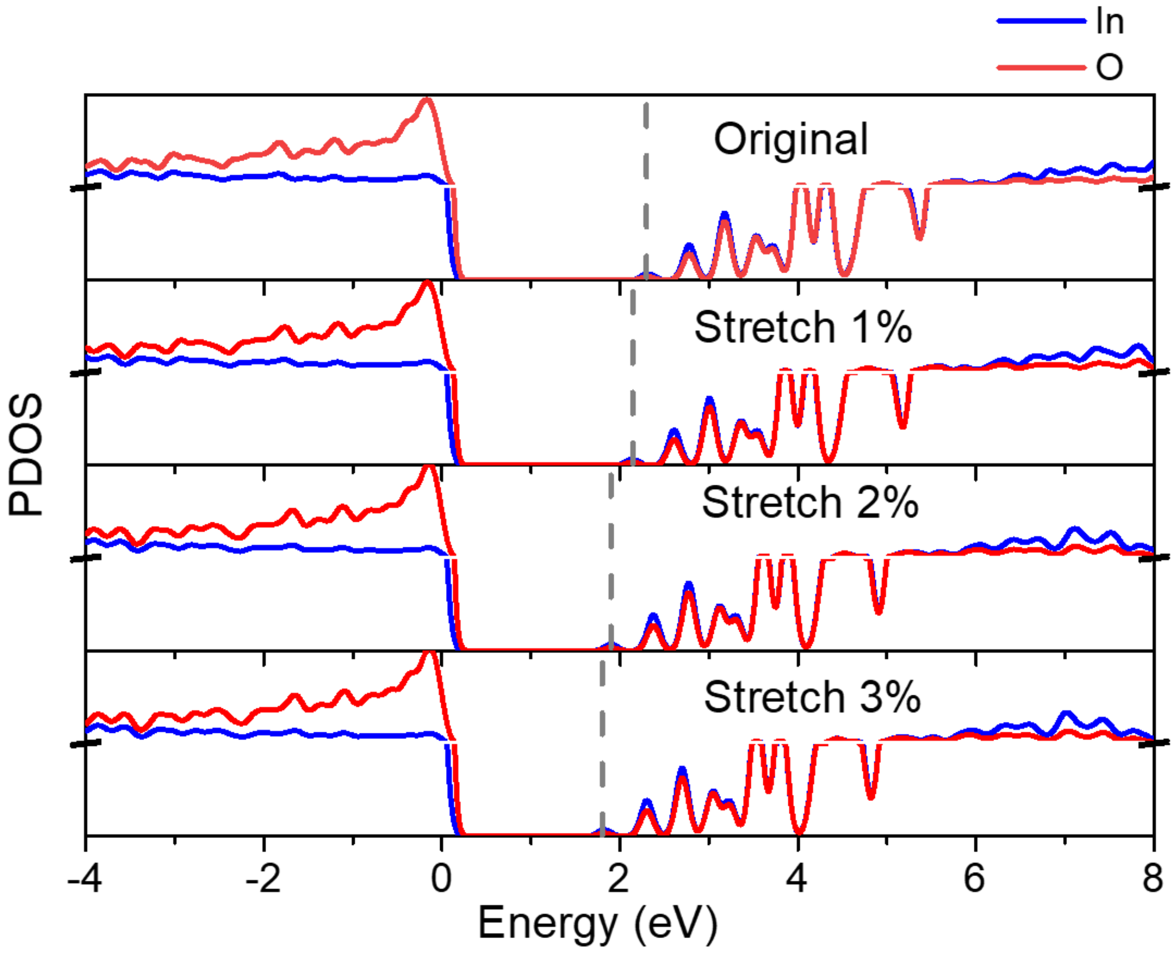

Supplementary Figure 12. Calculated PDOS of In_2O_3 crystals for different sizes. Blue and red lines show the contribution of In and O atoms respectively. Tails of valance band are set as the zero level. Vertical dashed lines can be helpful to find the shift of peaks. The calculated partial density of states (PDOS) with lattice parameter stretched up to 3%. Frontier valance bands of In_2O_3 are mainly contributed by O atoms while conduction bands have projection on both In and O atoms. The gap monotonically becomes smaller as the lattice parameter increases. The estimated bandgaps decrease monotonically from 2.31eV to 1.82eV with increasing lattice expansion.

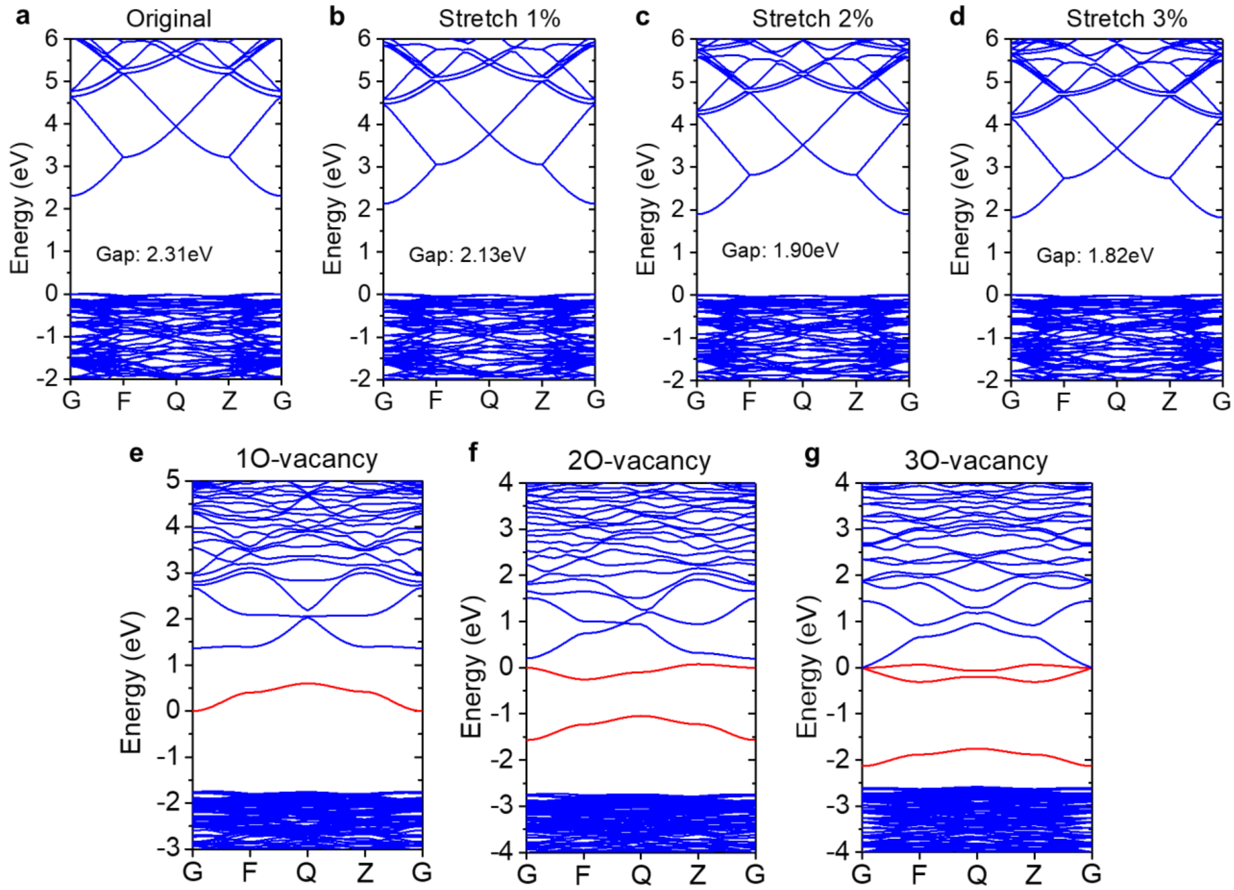

Supplementary Figure 13. Simulated band structure for In_2O_3 crystals. (a-d) Valence band maximums are set as the zero level. The width of the direct gap at gamma point is marked in each panel. The bandgap decreases from 2.31eV to 1.82eV when the lattice is stretched by 3%. (e-g) Calculated band structures for defective structures shown in Supplementary Figure 11b-d. Defect states in red are found in the middle of the bandgaps, which may enhance the adsorption of light.

.

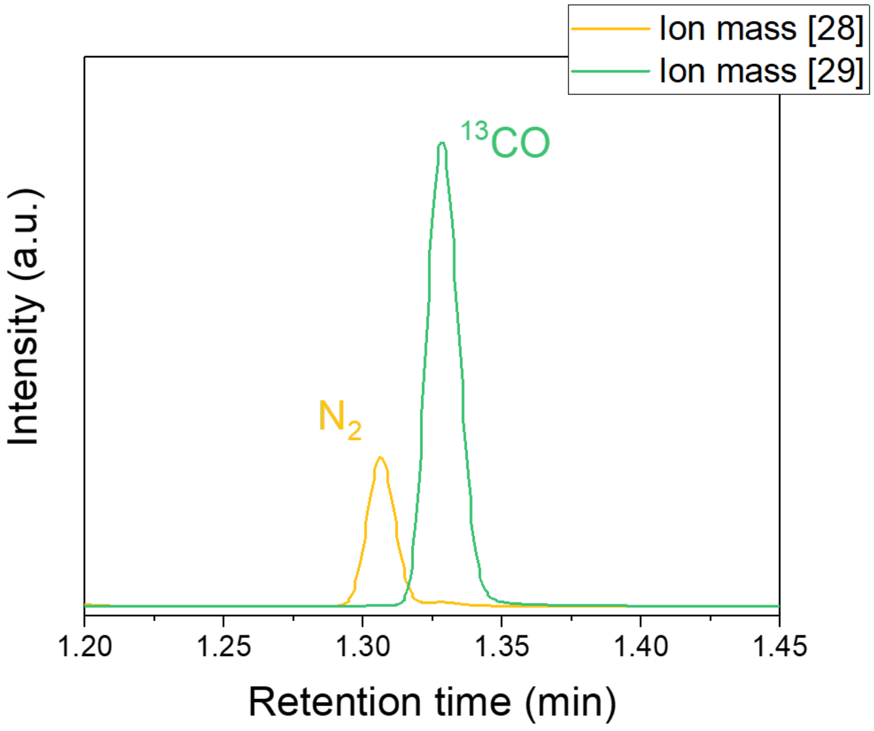

Supplementary Figure 14. Mass spectroscopy of S4 generated ^{13}CO from $^{13}CO_2$. The 28 AMU mass fragment peak at approximately 1.32 min corresponds to adventitious N_2 and the 29 AMU mass fragment peak at approximately 1.35 min corresponds to ^{13}CO. The fact that there is no peak near 1.35 min retention time for the 28 AMU curve shows that there is no ^{12}CO in the products generated from sources of adventitious ^{12}C contamination.

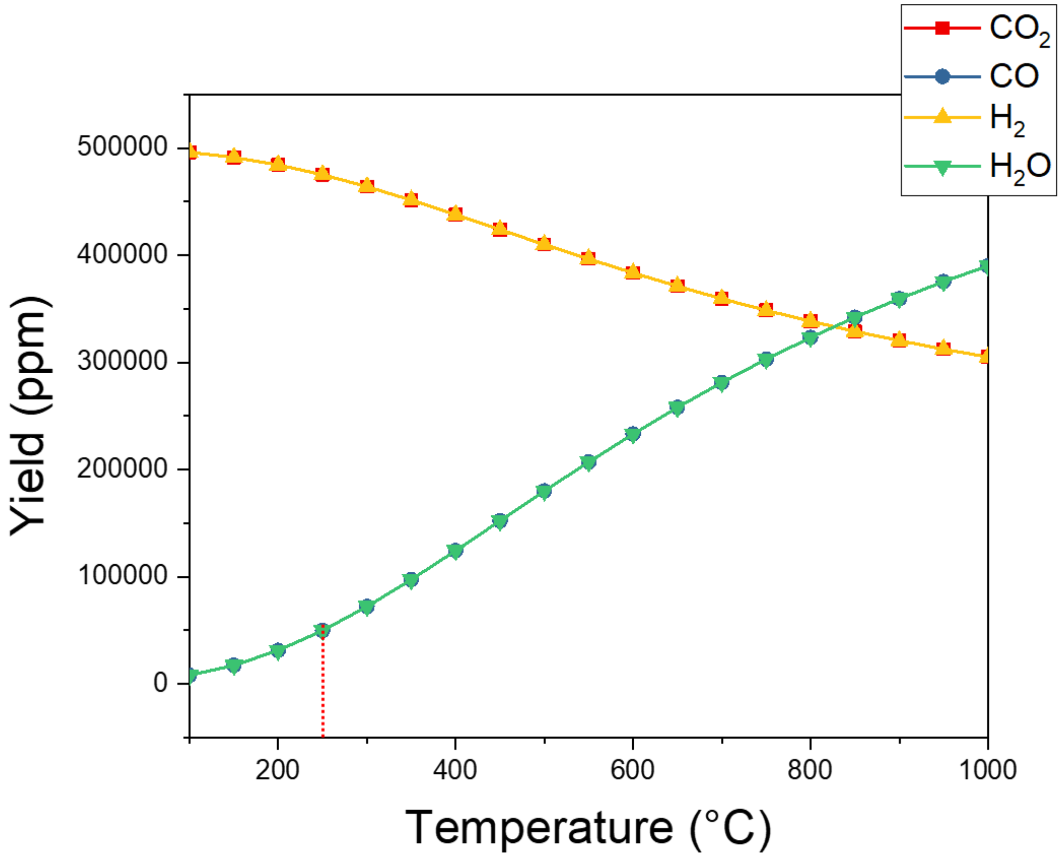

Supplementary Figure 15. Thermodynamic equilibrium composition as a function of temperature for the reverse water gas shift reaction at conditions of 1:1 H_2:CO_2 and 9.2 psi pressure. Furthermore, the yield of S1 to S3 are 18, 24 and 50 ppm and indicate much lower local temperatures (less than 100 ˚C) than S4 with 55061 ppm (262 ˚C).

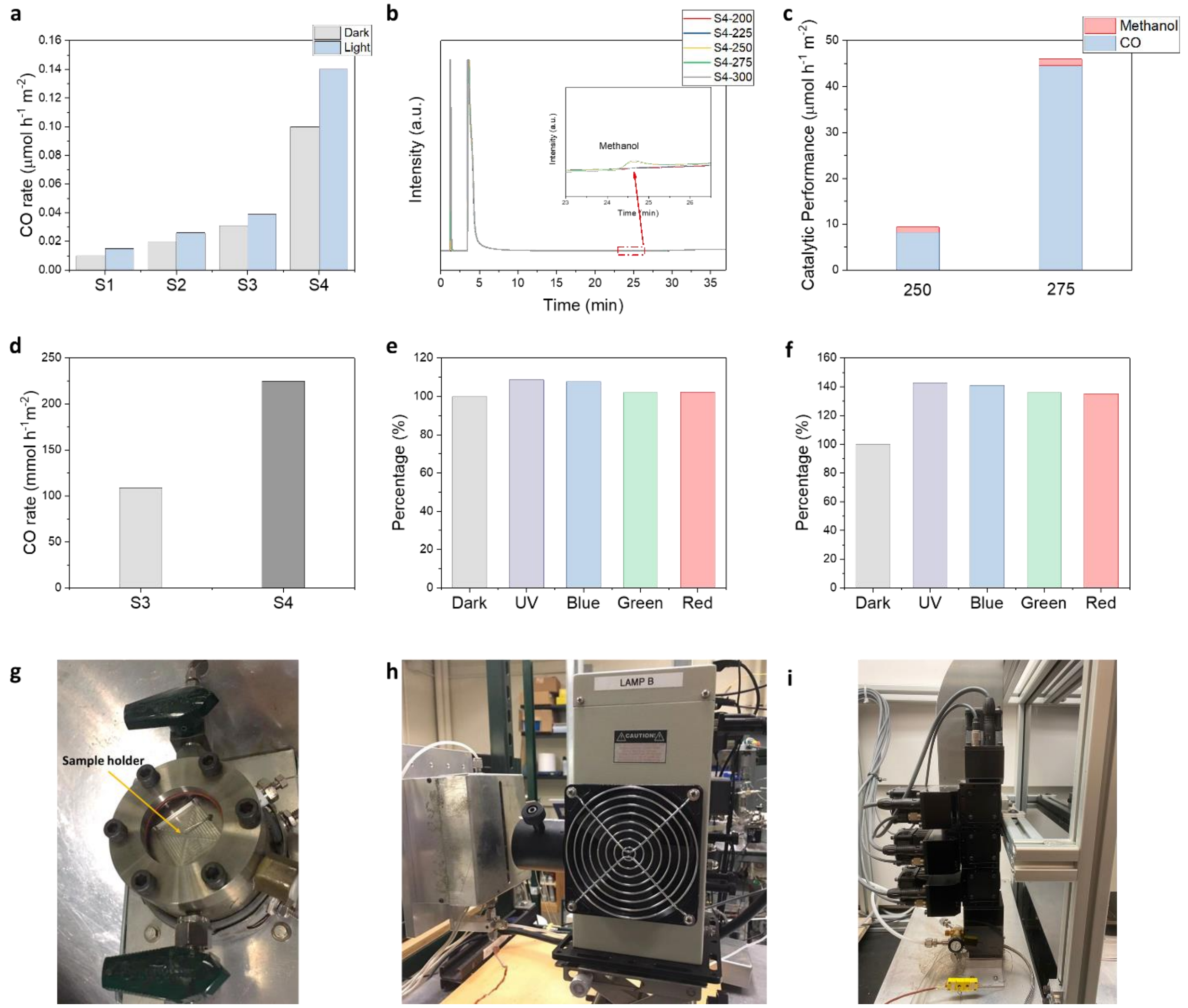

Supplementary Figure 16. (a) Catalytic performance of S1 to S4 in a flow reactor at 200 ˚C. (b) GC spectra of S4 at different temperatures with light irradiation and (c) Photocatalytic performance of S4 at 250 ˚C and 275 ˚C for both CO and methanol. (8.27 and 44.56 μmol $h^{-1}m^{-2}$ for CO; 1.14 and 1.48 μmol $h^{-1}m^{-2}$ for methanol) (d) Catalytic performance of S3 and S4 at 300 ˚C in LED flow reactor without any light irradiation. Photoaction behavior of (e) S3 and (f) S4. Test conditions: 300 ˚C, light intensity of ~5 suns, pressure of ~40 psi, gas ratio CO_2:H_2 = 1:1 at 2 ml/min. The peak wavelengths of UV, blue, green and red LED sources are 365, 440, 525 and 625 nm, respectively. Pictures of reactor setup (g) batch reactor, (h) flow reactor and (i) LED reactor.

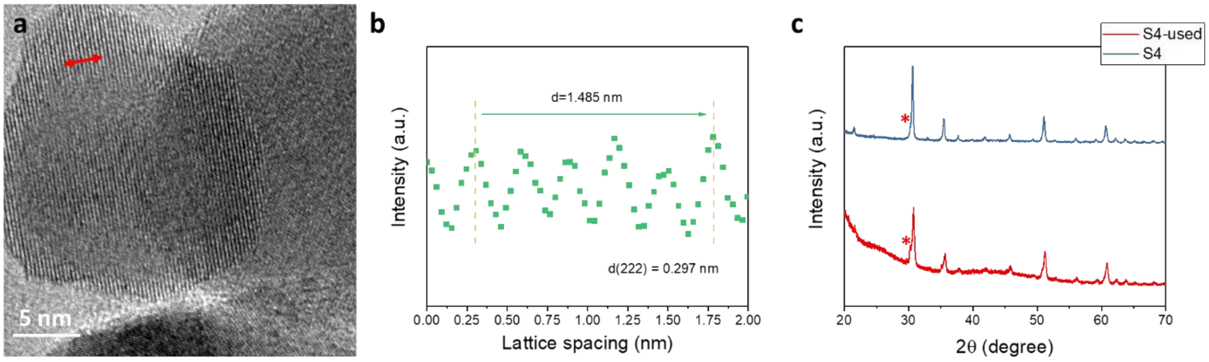

Supplementary Figure 17. (a) HRTEM, (b) normalized pixel intensities for (222) facets, the peaks and valleys represent regions of high and low electron contrast, respectively, and (c) PXRD patterns of the spent S4.

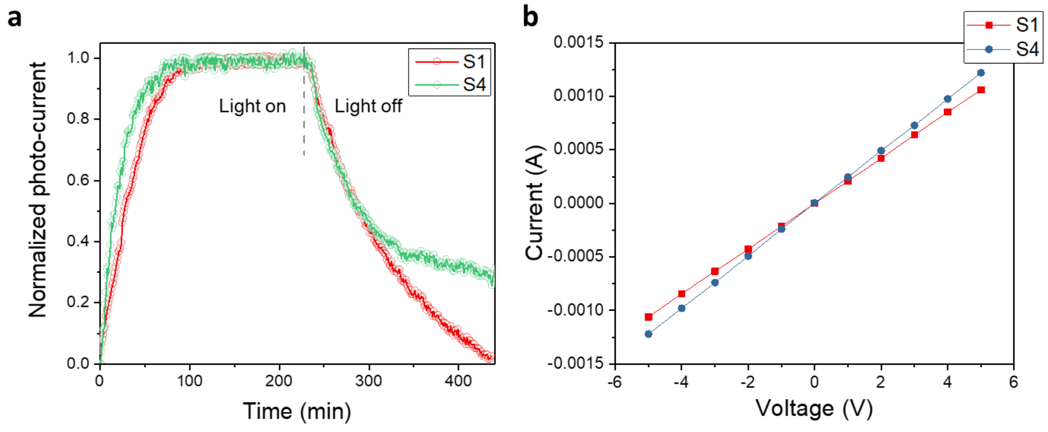

Supplementary Figure 18. (a) *In situ* photoconductivity measurement of S1 and S4 under vacuum condition at room temperature. (b) The corresponding I-V plot for S1 and S4.

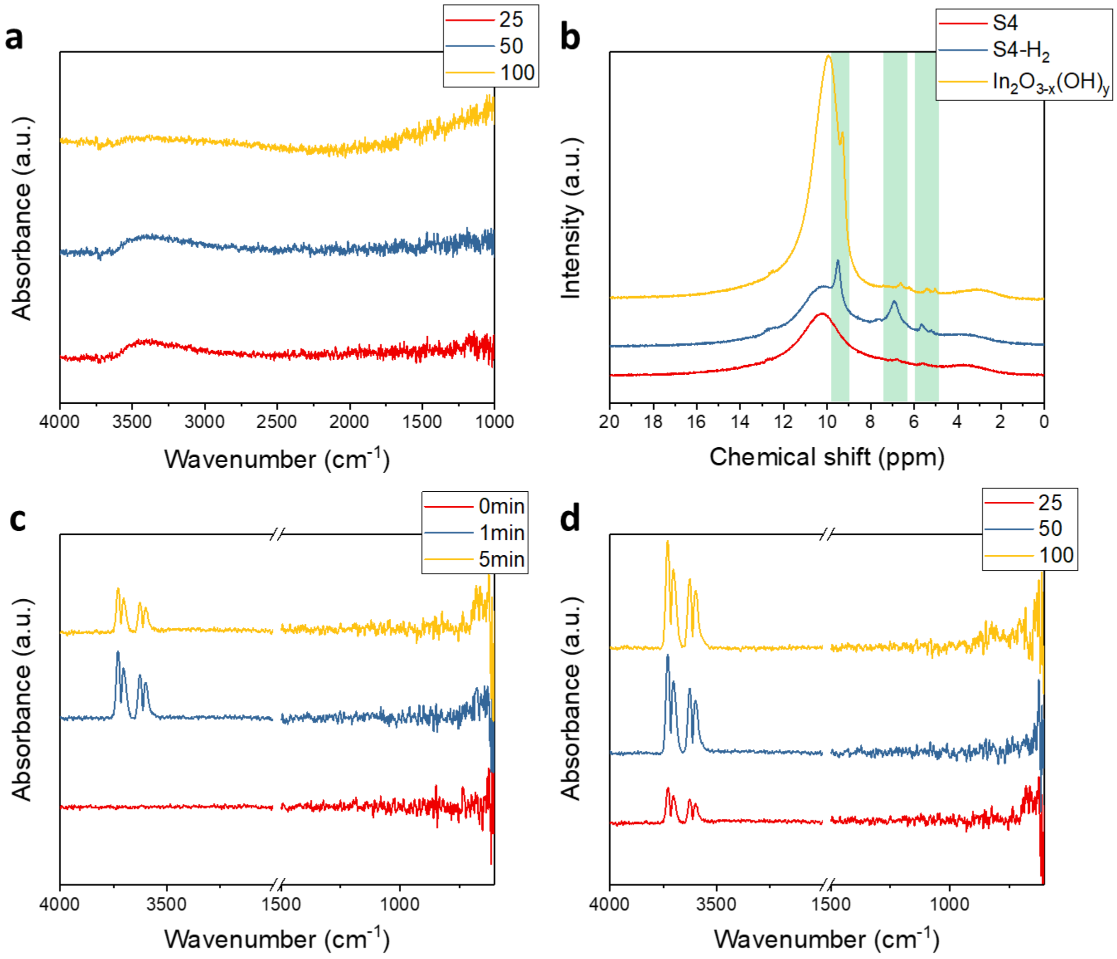

Supplementary Figure 19. (a) *Operando* DRIFTS for S4 under H_2 at different temperatures and (b) ^1H solid state MAS NMR spectroscopy for S4 before and after exposure to H_2 at room temperature and $In_2O_{3-x}(OH)_y$ without any treatment. *Operando* DRIFTS for S4 (c) under CO_2 at room temperature, and (d) under CO_2 with increasing temperatures.

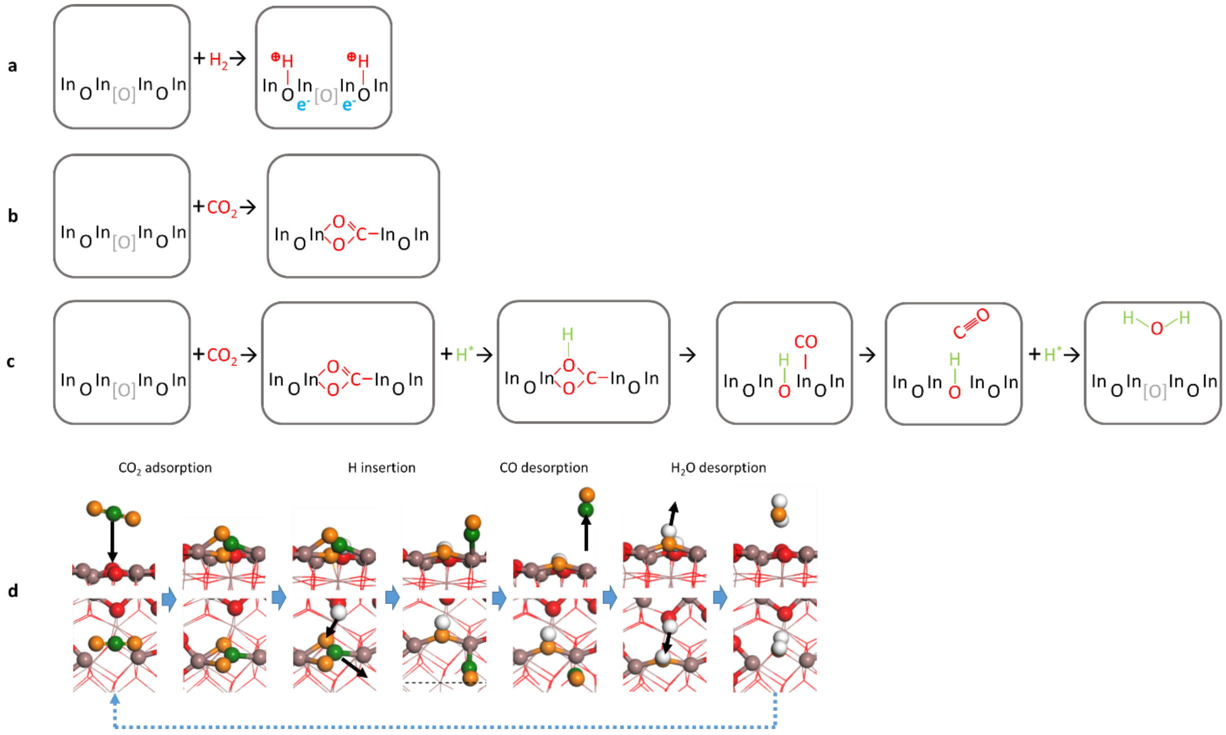

Supplementary Figure 20. Proposed reaction pathways for (a) H_2 dissociation, (b) CO_2 adsorption, (c) CO_2 hydrogenation and (d) stimulated CO_2 hydrogenation pathway. Energy barriers for H insertion, CO desorption and H_2O desorption are 1.31eV, 0.38 eV and 1.87 eV respectively. The CO_2 adsorption process is exothermic with energy decrease of 0.84eV.

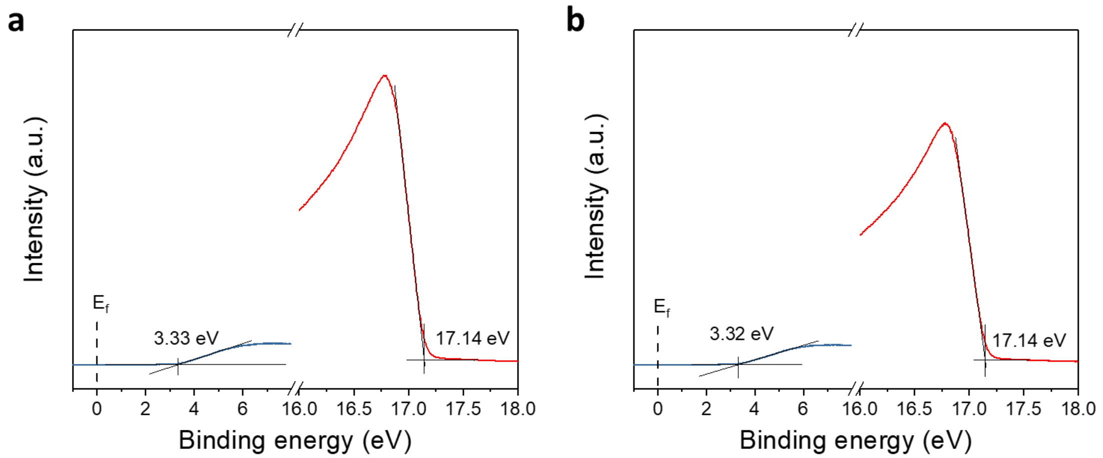

Supplementary Figure 21. UPS measurement of S1 (a) and S4 (b) establish conduction band and valence band positions of -4.73 eV and -7.39 eV for S1; and -5.02 eV and -7.38 eV for S4.

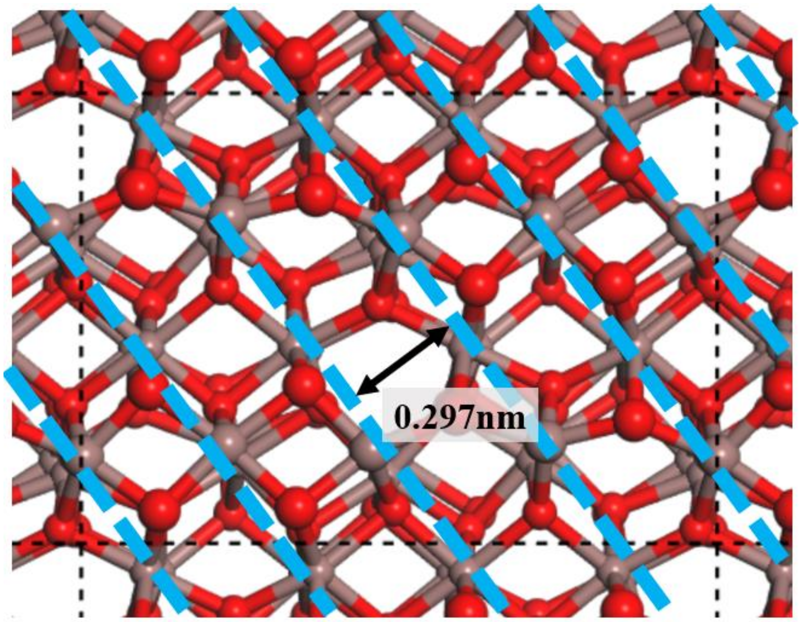

Supplementary Figure 22. Top view of In_2O_3 (110) surface. Red and brown spheres represent O and In atoms respectively. (222) facets are marked by blue dashed lines. The average distance between adjacent (222) facets is calculated to be 0.297 nm for a defect-free surface, consistent with the measured lattice spacing. This lattice spacing confirms that the surface of the sample is In_2O_3(110).

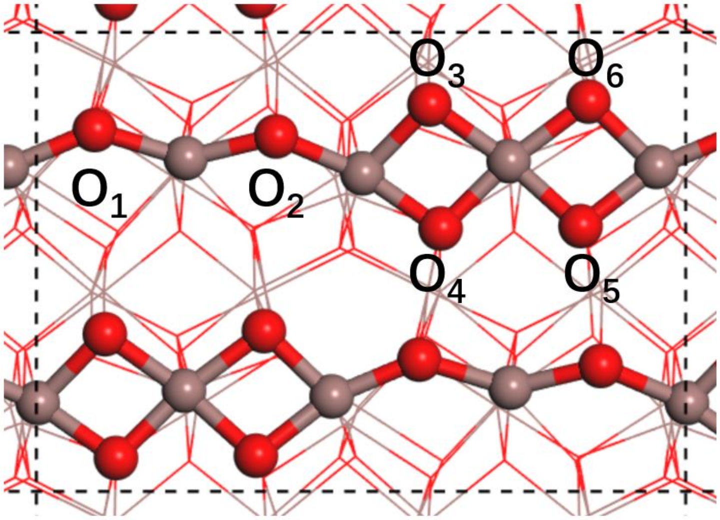

Supplementary Figure 23. Top view of simulated atomic structure for the In_2O_3 (110) surface. The structure is the same to the Supplementary Fig. 22. Candidate [O] vacancy sites are marked as O_1 to O_6. O_1 is used to simulate the reaction pathway.

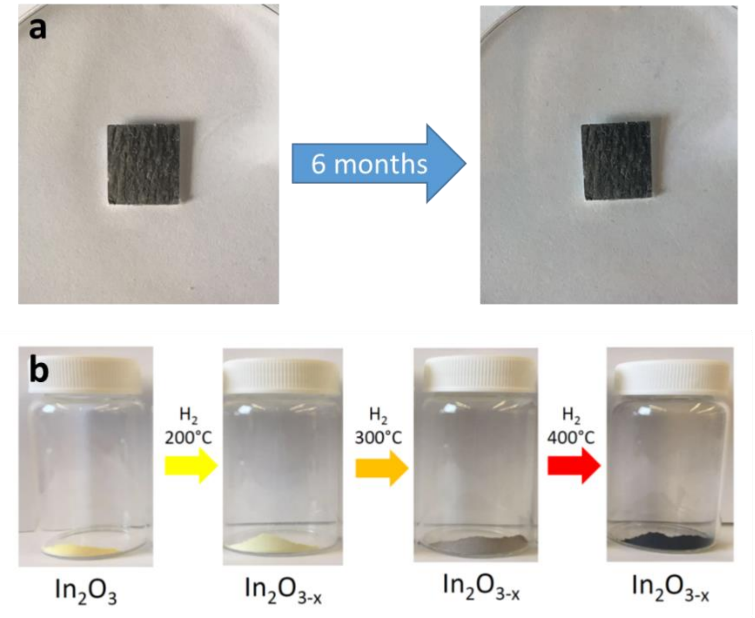

Supplementary Figure 24. (a) As-prepared black indium oxide stay in ambient condition for more than 6 months. (b) Color changes from pale yellow to grey to black observed on hydrogenating stoichiometric In_2O_3 at 200, 300, 400°C to form non-stoichiometric In_2O_{3-x} with monotonically increasing values of x with temperature.

Supplementary Table 1. Calculated turnover frequency for CO_2 hydrogenation catalysts, including photothermal catalysts, photocatalysts and thermal catalysts.

**Materials**	**TOF (s^{-1})**	**Catalytic Method**	**Reference**
Black indium oxide	2.12	Photothermal	This work
Single atom Ni/Y_2O_3	0.023	Photothermal	*3*
Pd/SiH nanosheet	0.09	Photochemical	*4*
$PtCo/TiO_2$	0.59	Thermal	*5*
$PtCo/CeO_2$	0.33	Thermal	*5*
$PtCo/ZrO_2$	0.26	Thermal	*5*
Pt_3Co octapods	0.21	Thermal	*6*
$5\%Pt/Al_2O_3$	0.02	Thermal	*7*
Ni/SiO_2	1.61	Thermal	*8*
NiFe	5.9	Thermal	*9*
Pd/Nb_2O_5	0.61	Photothermal	*10*

Supplementary Table 2. Structural parameter values obtained via fitting of Fourier-transformed EXAFS spectra, including coordination numbers (CN) and bond length (R). Numbers in parenthesis represent the 1σ uncertainty in the last decimal place of the preceding value.

Sample	Path	CN	R (Å)	σ^2 (10^{-3} Å^2)	ΔE_0 (eV)
S1	In-O	6.0(3)	2.165(4)	6.5(7)	-0.6(4)
	In-In	5.2(5)	3.354(3)	4.4(5)	
	In-In	5(1)	3.838(6)	6(1)	
S1 (Reduced, 2 h)	In-O	5.8(2)	2.169(4)	6.2(6)	-0.8(3)
	In-In	5.1(4)	3.358(2)	4.3(3)	
	In-In	5.1(8)	3.843(4)	6.2(8)	
S1 (Reduced, 4 h)	In-O	6.0(3)	2.168(4)	6.4(7)	-0.1(3)
	In-In	5.2(5)	3.358(3)	4.4(4)	
	In-In	5.1(9)	3.843(5)	6(1)	
S4	In-O	5.3(4)	2.164(6)	6(1)	-0.4(5)
	In-In	4.7(6)	3.353(4)	4.1(6)	
	In-In	5(1)	3.840(7)	6(1)	

Supplementary Table 3. Simulated In-In distance with different number of removed O atoms.

Number of removed O atoms	d1 (nm)	d2 (nm)	d3 (nm)
Crystalline	0.391	0.391	0.391
1	0.391	0.390	0.388
2	0.381	0.366	0.366
3	0.358	0.358	0.358

Supplementary Table 4. Calculated thermal desorption energies for O_1-O_6 atoms on the model In_2O_3 (110) surface and the reaction energy for CO_2 + In-[O]-In' → CO + In-O-In and H_2 + In-O-In → In-[O]-In' + H_2O. Definitions for ΔE_1 and ΔE_2 are $\Delta E_1 = E_{\mathrm{CO}} + E_{\mathrm{In_2O_3(110)}} - E_{\mathrm{CO_2}} - E_{\mathrm{In_2O_3(110)Ovac}}$ and $\Delta E_2 = E_{\mathrm{H_2O}} + E_{\mathrm{In_2O_3(110)Ovac}} - E_{\mathrm{H_2}} - E_{\mathrm{In_2O_3(110)}}$. Values are consistent with literature values[11]. Thermal desorption energies of surface oxygen are 2.01 eV to 2.58 eV. Notably, if the oxygen instead is abstracted by H_2, the reaction energies drop to -0.53 eV to 0.04 eV, supporting the proposal of more facile O vacancy formation in a H_2-rich environment. Calculated reaction energies for CO_2 hydrogenation range from 0.69 eV to 1.26 eV. The reaction can therefore happen under solar irradiation conditions.

	**Thermal desorption (eV)**	**ΔE_1 (eV)**	**ΔE_2 (eV)**
O_1	2.58	0.69	0.04
O_2	2.43	0.84	-0.11
O_3	2.01	1.26	-0.53
O_4	2.06	1.21	-0.48
O_5	2.40	0.86	-0.13
O_6	2.14	1.13	-0.40

Supplementary Methods

Calculation of turnover frequency

$$TOF = \frac{number\ of\ produced\ molecues}{number\ of\ active\ sites * total\ reaction\ time\ (s)} \qquad \text{(Supplementary Equation 1)}$$

$N_A = 6.022 \times 10^{17}\ \mu mol^{-1}$

Number of produced molecules per second = CO rate ($\mu mol\ h^{-1}m^{-2}$) x N_A / total reaction time

Total reaction time = 3600 s

S4 rate: 1874.62 $\mu mol\ h^{-1}m^{-2}$

The number of produced molecules per second for S4 is $3.14 \times 10^{17}\ m^{-2}s^{-1}$

As shown in **Supplementary Fig. 23**, the simulated exposed facet has 12 surface oxygen atoms, where red and brown colors denote oxygen and indium atoms.

Area of simulated exposed facet 14.57Å x 10.31 Å = $1.50 \times 10^{-18}\ m^2$

Number of surface O atoms per square meter = $8 \times 10^{18}\ m^{-2}$

Therefore the overall surface O atoms can be calculated by multiplying the number of O atoms per square meter with the specific surface area obtained from BET measurements.

Model 1: Assume all [O] will be used for reaction

Number of active sites = number of [O] = $8 \times 10^{18}\ m^{-2}$ x [O] value obtained from XPS (37.34%) = $2.99 \times 10^{18}\ m^{-2}$

$TOF_{S4} = 3.14 \times 10^{17}\ m^{-2}s^{-1} / 2.99 \times 10^{18}\ m^{-2} = 0.10\ s^{-1}$

Similarly, $TOF_{S1} = 5.64 \times 10^{-5}\ s^{-1}$, $TOF_{S2} = 1.17 \times 10^{-4}\ s^{-1}$ and $TOF_{S3} = 1.77 \times 10^{-4}\ s^{-1}$.

Model 2: Assume only extrinsic [O] will be used for reaction

Number of active sites = number of extrinsic [O] = $8 \times 10^{18}\ m^{-2}$ x ([O] value obtained from XPS (37.34%) – intrinsic [O] of In_2O_3 (25%)) = $0.99 \times 10^{18}\ m^{-2}$

$TOF_{S4} = 3.14 \times 10^{17}\ m^{-2}s^{-1} / 0.99 \times 10^{18}\ m^{-2} = 0.32\ s^{-1}$

As a result, $TOF_{S1} = 4.17 \times 10^{-4}\ s^{-1}$, $TOF_{S2} = 5.49 \times 10^{-4}\ s^{-1}$ and $TOF_{S3} = 6.21 \times 10^{-4}\ s^{-1}$.

Model 3: Assume only extrinsic [O] mainly located in amorphous region will be used for reaction

Based on *in situ* HRTEM from Fig. 2d, the formed amorphous region covers roughly = ~43 nm^2 / ~330 nm^2 = ~13%

Number of active sites = $0.99 \times 10^{18}\ m^{-2}$ x 13% = $0.1287 \times 10^{18}\ m^{-2}$

$TOF_{S4} = 3.14 \times 10^{17}\ m^{-2}s^{-1} / 0.1287 \times 10^{18}\ m^{-2} = 2.44\ s^{-1}$

Characterization

PXRD was performed on a Bruker D2-Phaser X-ray diffractometer, using Cu Kα radiation at 30 kV. Nitrogen Brunauer-Emmet-Teller (BET) adsorption isotherms were obtained at 77 K using a Quantachrome Autosorb-1-C. Diffuse reflectance of the samples were measured using a Lambda 1050 UV-Vis-NIR spectrometer from PerkinElmer and an integrating sphere with a diameter of 150 mm. XPS was performed using a PerkinElmer Phi 5500 ESCA spectrometer in an ultrahigh vacuum chamber with base pressure of 1×10^{-9} Torr. The spectrometer uses an Al Kα X-

ray source operating at 15 kV and 27 A. The samples were coated onto carbon tape, and all results were calibrated to C1s 284.5 eV. UPS was performed on a Thermo Fisher Scientific EscaLab 250 Xi with He I (21.22eV) for UPS characterization. Ag was used as reference material for later calibration for Fermi edge. The ^1H solid state MAS NMR spectra were obtained at a spinning rate of 12 kHz. The NMR Spectra were calibrated to reference adamantane with optimized parameters: pulse width (pwX90) = 3.45 microseconds, fine power (aX90) = 2700, course power (tpwr) = 59, synthesizer offset (tof) = 1192.9. Number of scans = 64, delay time = 6 s. Samples for ^1H solid state MAS NMR were treated by H_2 and then transferred to a glovebox with an Ar atmosphere for sample loading. Raman spectroscopy was performed using a JY Horiba LabRam HR800 Raman instrument. The excitation laser has a wavelength of 632.817 nm. The measurements were carried out using an objective lens with a magnification of 100x, a grating of 1800 lines/mm, a spot size of 965 nm and a hole size of 100 µm with an irradiation area diameter of 140 µm. The light intensity was measured with a Spectra-Physics Power meter (model 407A). The measured reading is about 2 W with a 1 cm^2 mask and giving a light intensity of 2 W/cm^2. It is known that 1 sun = 0.1 W/cm^2, thus the light intensity is about 20 suns in the batch reactor. The same method is applied to the flow reactor and the light intensity is calculated as 8 suns. Thermogravimetric measurements were performed using a TA instruments Discovery TGA. The flow rate was kept steady at 20 mL/min. A heating rate of 10 ˚C /min was used for the sample heated to 700 C in air.

<u>Estimation of local temperature</u>

The local temperature of the catalyst was estimated under illumination of ~20 suns. By allowing the system to approach equilibrium we used the final dry basis gas composition to estimate the temperature by comparing with Aspen Plus V9 output. We made use of the Peng-Robinson property package for the equilibrium analysis with the Gibbs reactor. The components under consideration were those involved in the reverse water gas shift reaction. The sample is characterized using Hitachi HF-3300 TEM, an environmental TEM working at an operating voltage of 300 kV.

<u>Estimation of x value of In_2O_{3-x}</u>

Based on the fitting profile of core level O 1s XPS spectra, the concentration of oxygen vacancy over the surface S2-S4 are 31.74%, 34.97% and 37.34%, respectively. Given that the stoichiometric In_2O_3 has 25% of intrinsic [O], and the resulting extrinsic [O] of S2-S4 are 6.74%, 9.97% and 12.34%.

In_2O_3 consists of 82.702 wt% In and 17.298 wt% O with a molar ratio of 2:3.

Thus for In_2O_{3-x}, the x value can be calculated as follow

$$\frac{17.298}{3} = \frac{17.298 * (100\% - extrinsic\ [O]\ of\ the\ sample)}{3 - x}$$

The x value for S2 to S4 can be calculated as 0.20, 0.30 and 0.37, respectively.

As a result, S2 is $In_2O_{2.8}$, S3 is $In_2O_{2.7}$ and S4 is $In_2O_{2.63}$.

<u>*In situ* X-ray absorption near-edge structure</u>

X-ray absorption near-edge structure (XANES) and extended X-ray absorption Fine Structure (EXAFS) at In K-edge (27940 eV) were measured, using a focused beam, at 20-BM-B of the

Advanced Photon Source at Argonne National Laboratory. A Si (111) fixed-exit, double-crystal monochromator was used and harmonic rejection was facilitated by detuning the beam intensity 15% at ~1000 eV above the edge of interest. Data was collected in transmission mode using N_2 in the I0 and 20%N_2 and 80%Ar in IT and Iref. Details on the beamline optics and instruments can be found elsewhere[12]. The sample (40mg+60mg BN) was prepared as pellet and placed in a furnace with Kapton® windows[13].
EXAFS measurements were performed for S1. The sample was exposed to 3.5%H_2/He (50 mL min^{-1}) and heated up to 400 °C (5 °C min^{-1}) for 2h. XANES was recorded during the heating and isothermal conditions. After 2h, the sample was cooled down to room temperature, under He, and EXAFS was measured. Since small changes were observed, the sample was reduced again, using the same conditions but for 4h. XANES was also recorded and at the end of the treatment the atmosphere was changed to He, the temperature was reduced and EXAFS measured at room temperature. EXAFS ex situ measurements were also performed for the S4.
Normalization of XANES spectra was performed using Athena, part of the Demeter software package[14]. Subsequent data processing and EXAFS fitting were performed using WinXAS software[15]. Scattering paths using in EXAFS fitting were calculated using FEFF8 software[16] in conjunction with an In_2O_3 structural model obtained from the Crystallographic Open Database[17]. A total of three scattering paths were using in fitting the EXAFS data: one corresponding to In–O nearest-neighbour interactions and two corresponding to longer-distance In–In scattering interactions.

In situ high resolution environmental transmission electron microscopy

The sample was characterized in Hitachi H-3300 TEM, an environmental transmission electron microscope, equipped with a gas injection system and a heating system. This is done through the use of a special TEM holder that has ports for both gas connection as well as heating system. The holder consists of a MEMS heating device. During heating, controller runs current through the micro coil and measures the resistance. A software namely Blaze converts this resistance measurements to obtain the actual temperature of the sample under experimental conditions. The sample is heated to 400 ˚C in N_2 before switching it to H_2. Gas pressures of 1 Pa was used during the H_2 and N_2 flow.

In situ diffuse reflectance infrared Fourier-transform spectroscopy

In situ DRIFTS measurements were performed to detect the possible surface reaction intermediates over S4 under different conditions. The spectra were collected using a Fourier-transform infrared spectroscopy spectrometer (Thermo, Nicolet 6700) equipped with an MCT detector. Before measurement, the catalyst was purged with He at 300 °C for 2h. The catalyst was subsequently cooled down to the selected temperature. The background spectrum with a resolution of 4 cm^{-1} was obtained at different selected temperature in He atmosphere. Then the catalyst was exposed to a CO_2/He, H_2/He or CO_2/H_2/He mixture (2 mL/min CO_2, 2 mL/min H_2 and 16 mL/min He) for 30 min. The *in situ* DRIFT spectra were recorded by collecting 32 scans at 4 cm^{-1} resolution.

In situ photoconductivity measurement

Photoconductivity measurements utilized a white LED light source on a pressed powder film of the sample, weighing ~1 mg, with electrical contact made between two evaporation deposited 500 nm thick gold electrodes on a glass substrate separated by a distance of 2 mm. The I-V plot of the samples in the reactor was measured under the conditions of CO_2:H_2, pressure ratio =1:1, ~200 ˚C and vacuum (2E-2 mbarr) at room temperature.

Photocatalytic performance

The gas phase photothermal catalytic measurements were conducted in a custom-built 1.5 mL stainless steel batch reactor with a fused silica view port sealed with Viton O-rings. The reactor was evacuated using an Alcatel dry pump prior to being purged with the reactant H_2 gas (99.9995%) at a flow rate of 15 mL min^{-1}. After purging the reactor, it was filled with a 1:1 stoichiometric mixture of H_2 (99.9995%) and CO_2 (99.999%) until the total pressure reached 30 psi. Reactors were irradiated with a 300 W Xe lamp for a duration of 0.5 h without external heat. Product gases were analyzed using flame ionization and thermal conductivity detectors installed in a SRI-8610 gas chromatograph equipped with a 3 in. Mole Sieve 13a and 6 in. Haysep D column. Isotopically labeled tracing experiments were performed using $^{13}CO_2$ (99.9 at%, Sigma-Aldrich). Isotope distributions in the product gases were measured using an Agilent 7890A gas chromatograph-mass spectrometer with a 60 m GS-carbon plot column leading to the mass spectrometer.

Gas-phase flow reactor measurements were carried out in a fixed-bed tubular reactor. The reactor housing was a borosilicate tube (3 mm outer diameter and 2 mm inner diameter). Within the reactor tube, ~20 mg of catalyst was packed between two portions of quartz wool, which supported the packed catalyst bed in the center. External heating was provided by conduction via a heated copper block surrounding the tubular reactor on all but one side. An OMEGA CN616 6-Zone temperature controller managed the temperature utilizing a thermocouple placed near the catalyst bed. Each temperature set point was maintained for 5 h (6 runs) before increasing it to the next set point. The dark and light conditions are tested in different temperature ramping. During the reaction, H_2 (Praxair 99.999%) and CO_2 (Praxair 99.999%) were flowed in a 1:1 ratio at a total volumetric flow rate of 2 sccm. For photocatalytic rate measurements, the reactor was irradiated with a 300 W Newport Xe lamp. An 8610 SRI Gas Chromatograph (10 Mol Sieve 5a column and a 60 Haysep D column) was used to identify and quantify product gases.

Gas-phase LED photoreactor measurements were performed in a stainless steel flow reactor with a fused silica view port sealed with Viton O-rings. The reactor is equipped with an array of LED beams, including UV (UHP-T-365-MP, peak wavelength at 365 nm), blue (UHP-T-460-DI, peak wavelength at 440 nm), green (UHP-T-520-DI, peak wavelength at 525 nm), and red (UHP-625-DI, peak wavelength at 625 nm). The adjust flow rate of CO_2 and H_2 are 2 min/ml and with a pressure of ~40 psi. The reaction products were tested with an online SRI 8610c gas chromatographer.

Theoretical Calculations

Simulations were conducted using the Vienna ab-initio simulation package (VASP)[18] using the density functional theory. The wavefunction for electrons are simulated using a plane wave basis set combined with the projector augmented wave method[19,20]. Energy cutoff for the basis set was set to 400 eV. The exchange correlation energy was calculated using the PBE functional

in general gradient approximation scheme[21], accompanied with a semi-empirical dispersion correction[22]. Since PBE functional usually underestimate the bandgap, corrections for Coulombic potentials should be considered. The applied U for the GGA+U method is 11eV for In-s orbitals and 8eV for O-p orbitals. The optimized lattice parameter decreases by only 0.5% after U corrections are implemented. Eighty atoms are used to simulate the conventional cell of cubic In_2O_3. The first Brillouin zone of the cell is sampled by a 5×5×5 k-mesh. Each model was fully relaxed until the residual force for each atom is smaller than 0.01 eV/ Å.

Supplementary References

1 Wang L, Ghoussoub M, Wang H, Shao Y, Sun W, Tountas AA*, et al.* Photocatalytic Hydrogenation of Carbon Dioxide with High Selectivity to Methanol at Atmospheric Pressure. *Joule*, **2** 1382-1382 (2018).
2 Yan TJ, Wang L, Liang Y, Makaremi M, Wood TE, Dai Y*, et al.* Polymorph selection towards photocatalytic gaseous CO2 hydrogenation. *Nat Commun*, **10**, 2521 (2019).
3 Li, Y., Hao, J., Song, H., Zhang, F., Bai, X., Meng, X., Zhang, H., Wang, S., Hu, Y., Ye, J. Selective light absorber-assisted single nickel atom catalysts for ambient sunlight-driven CO2 methanation. *Nat. Commun.* **10**, 2359 (2019).
4 Qian, C., Sun, W., Hung, D. L. H., Qiu, C., Makaremi, M., Kumar, S. G. H., Wan, L., Ghoussoub, M., Wood, T. E., Xia, M., Tountas, A. A., Li, Y. F., Wang, L., Dong, Y., Gourevich, I., Singh, C. V., Ozin, G. A. Catalytic CO2 reduction by palladium-decorated silicon–hydride nanosheets. *Nat. Catal*. **2**, 46-54 (2019).
5 Kattel, S., Yu, W., Yang, Z., Yan, B., Huang, Y., Wan, W., Liu, P., Chen. J. G. CO2 Hydrogenation over Oxide-Supported PtCo Catalysts: The Role of the Oxide Support in Determining the Product Selectivity. *Angew. Chem. Int. Ed.* **55**, 7968-7973 (2016).
6 Khan, M. U., Wang, L., Liu, Z., Gao, Z., Wang, S., Li, H., Zhang, W., Wang, M., Wang, Z., Ma, C., Zeng, J. Pt3Co Octapods as Superior Catalysts of CO2 Hydrogenation, *Angew. Chem. Int. Ed*. **55**, 9548-9552 (2016).
7 Wang, X., Shi, H., Kwak, J. H., Szanyi, J. Mechanism of CO2 Hydrogenation on Pd/Al2O3 Catalysts: Kinetics and Transient DRIFTS-MS Studies, *ACS Catal*. **5**, 6337-6349 (2015).
8 Aziz, M. A. A., Jalil, A. A., Triwahyono, S., Mukti, R. R., Taufiq-Yap, Y. H., Sazegar, M. R. Highly active Ni-promoted mesostructured silica nanoparticles for CO2 methanation, *Appl Catal B.*, **147**, 359-368 (2014).
9 Pandey, D., Deo, G. Effect of support on the catalytic activity of supported Ni–Fe catalysts for the CO2 methanation reaction, *J. Ind. Eng. Chem*. **33**, 99-107 (2016).
10 Jia, J. Wang, H., Lu, Z., O'Brien, P. G., Ghoussoub, M., Duchesne, P., Zheng, Z., Li, P., Qiao, Q., Wang, L., Gu, A., Jelle, A. A., Dong, Y., Wang, Q., Ghuman, K. K., Wood, T., Qian, C., Shao, Y., Qiu, C., Ye, M., Zhu, Y., Lu, Z., Zhang, P., Helmy, A. S., Singh, C. V., Kherani, N. P., Perovic, D. D., Ozin, G. A. Photothermal Catalyst Engineering: Hydrogenation of Gaseous CO2 with High Activity and Tailored Selectivity, *Adv. Sci*. **4**, 1700252 (2017).
11 Ye J, Liu C, Mei D, Ge Q. Active Oxygen Vacancy Site for Methanol Synthesis from CO2 Hydrogenation on In2O3(110): A DFT Study. *Acs Catal* **3**, 1296-1306 (2013).
12 Heald, S. M. Optics upgrades at the APS beamline 20-BM. Nuclear Instruments and Methods in Physics Research, Section A: Accelerators, Spectrometers, Detectors and Associated Equipment, **649**, 128–130 (2011).
13 Bolin, T. B., Wu, T., Schweitzer, N., Lobo-Lapidus, R., Kropf, A. J., Wang, H., Hu, Y., Miller, J. T., Heald, S. M., In situ intermediate-energy X-ray catalysis research at the advanced photon source beamline 9-BM. *Catalysis Today*, **205**, 141–147 (2013).
14 Ravel, B., Newville, M. ATHENA, ARTEMIS, HEPHAESTUS: Data analysis for X-ray absorption spectroscopy using IFEFFIT. *J. Synchrotron Radiat*. **12**, 537-541 (2005).

15 Ressler, T., WinXAS: A program for X-ray absorption spectroscopy data analysis under MS-Windows. *J. Synchrotron. Radiat*. **5**, 118–122 (1998).
16 Ankudinov, A. L., Ravel, B., Rehr, J. J., Conradson, S. D., Real-space multiple-scattering calculation and interpretation of X-ray-absorption near-edge structure. *Phys. Rev. B,* **58**, 7565–7576 (1998).
17 Grazulis, S., Daskevic, A., Merkys, A., Chateigner, D., Lutterotti, L., Quiros, M., Serebryanaya, N. R., Moeck, P., Downs, R. T., Bail, A., Crystallography open database (COD): An open-access collection of crystal structures and platform for world-wide collaboration. *Nucleic Acids Res*. **40**, D420-D427 (2012).
18 G. Kresse, J. Furthmüller, Efficient Iterative Schemes for Ab Initio Total-Energy Calculations Using a Plane-Wave Basis Set. *Phys. Rev. B* **54**, 11169-11186 (1996).
19 G. Kresse, D. Joubert, From Ultrasoft Pseudopotentials to the Projector Augmented-Wave Method. *Phys. Rev. B* **59**, 1758-1775 (1999).
20 P. E. Blöchl, Projector Augmented-Wave Method. *Phys. Rev. B* **50**, 17953-17979 (1994).
21 J. P. Perdew, K. Burke, M. Ernzerhof, Generalized Gradient Approximation Made Simple. *Phys. Rev. Lett*. **77**, 3865 (1996).
22 S. Grimme, J. Antony, S. Ehrlich, S. Krieg, J. Chem. Phys. 132, 154104 (2012).
